# Structural and Antioxidant Comparison Between Native WPI and WPI-Resveratrol Non-Covalent Complex

**DOI:** 10.3390/antiox15091096

**Published:** 2026-08-31

**Authors:** Juexi Liu, Lingtong Fan, Jingran Wei, Qingsong Liu, Ouyan Han, Yan Yang, Danjun Guo, Wei Xu, Huajuan Wang, E Liao

**Affiliations:** 1 College of Food Science and Engineering, Wuhan Polytechnic University, Wuhan 430023, China; ljx4169161107@163.com (J.L.); fanlt2005@163.com (L.F.); 18871281393@163.com (J.W.); lqs2892013390@163.com (Q.L.); hanouyan20020711@163.com (O.H.); xuwei1216@163.com (W.X.); wanghuajuan0609@126.com (H.W.); liaoe1987@whpu.edu.cn (E.L.); 2 Hubei Key Laboratory for Processing and Transformation of Agricultural Products, Wuhan 430023, China

**Keywords:** resveratrol, whey protein isolate, non-covalent binding, antioxidant, molecular dynamics simulation

## Abstract

Population aging has made sarcopenia a growing concern in geriatric health. Protein–polyphenol non-covalent complexes can serve as carrier systems that improve the stability and bioactivity of natural antioxidants. This study refined the preparation parameters for the non-covalent complex of whey protein isolate (WPI) and resveratrol (RES), achieving protein digestibility of 83.60 ± 0.50% and DPPH scavenging of 52.41 ± 0.58% at pH 7.0, a WPI:RES molar ratio of 1:1, and a reaction time of 1.5 h. Relative to free WPI, the complex improved DPPH scavenging by 21.66% while preserving protein digestibility. The binding mode, interaction forces, and conformational evolution were investigated via spectroscopic experiments, docking studies and molecular dynamics simulations. RES selectively bound to the surface of β-lactoglobulin in WPI through hydrophobic interactions and hydrogen bonding, with a docking score of −6.366 kcal/mol and an MM-GBSA binding free energy of −30.48 kcal/mol. The complex maintained a highly stable conformation throughout the 100 ns molecular dynamics simulation. In conclusion, this study elucidated the structural changes of WPI upon non-covalent resveratrol binding. The WPI-RES complex exhibited enhanced DPPH radical scavenging activity while preserving protein digestibility, with potential implications for functional food development in sarcopenia management.

## 1. Introduction

With progressive population aging, sarcopenia has emerged as a major threat to the health of older adults. Marked by losses in muscle mass, strength, and function, this condition hinders daily activities and may cause metabolic disorders [1]. The pathogenesis involves muscle protein imbalance, mitochondrial dysfunction, and oxidative stress [2]. Dietary components including proteins, polyphenols, and other bioactive nutrients can alleviate sarcopenia by modulating muscle metabolism and reducing inflammation [3]. In recent years, dietary nutritional intervention has become a core strategy for the prevention and treatment of sarcopenia, and related research and applications have attracted considerable attention [4]. Among these dietary components, proteins and polyphenols have gradually become a research focus in this field because of their distinct physiological functions and potential synergistic effects.

Key amino acids such as leucine and glutamine, released upon protein digestion, can act as crucial nutrient-signaling molecules to activate the mTOR signaling pathway in skeletal muscle, thereby positively regulating muscle protein anabolism. Whey protein isolate (WPI) is widely used in special dietary products due to its high digestibility and rich content of branched-chain amino acids [5]. However, WPI is prone to denaturation and aggregation during processing, which compromises its functionality and bioactivity.

Polyphenols can effectively improve skeletal muscle function through anti-inflammatory, antioxidant, and cytoprotective mechanisms. They inhibit protein degradation via the Akt/mTOR axis and enhance mitochondrial function through AMPK/SIRT1 activation [6]. In addition, polyphenols can efficiently scavenge excessive reactive oxygen species in skeletal muscle, suppress the NF-κB pathway and downstream inflammatory cytokine release, and thus alleviate oxidative stress and chronic low-grade inflammation-induced damage to muscle cells [7]. Resveratrol (RES), a plant-derived polyphenol, exhibits strong antioxidant and anti-inflammatory activities that effectively slow apoptosis.

Thus, efficient binding of proteins and polyphenols to harness their synergistic benefits is now a major research priority. The binding between proteins and polyphenols is primarily categorized as non-covalent or covalent. Non-covalent binding, being dynamic and reversible, is the most basic interaction in food systems and sees broad use in the food industry. Shu et al. [8] reported that rice protein, after non-covalent binding with polyphenols, exhibited improved emulsifying properties and solubility, and the synergistic effect with polyphenols greatly inhibited lipid oxidation. Cao et al. [9] found that, compared with free polyphenols, BSA-polyphenol non-covalent complexes showed a substantially higher DPPH radical scavenging rate and a 2–3-fold extended half-life of antioxidant activity, effectively preventing the loss of polyphenol bioactivity. Based on numerous studies, non-covalent protein–polyphenol complexation improves protein functionality and polyphenol bioactivity, thus helping to overcome the limits of single components.

In this study, WPI and RES were chosen to construct a non-covalent complex. Most studies focus on the properties of protein–polyphenol complexes alone, but direct comparisons between the complexed form and the unmodified protein—especially at the molecular level—are still limited. So, we optimized the preparation conditions to get a product with good antioxidant activity and digestibility, then used multi-spectroscopic techniques and molecular simulations to compare the structural and functional differences between free WPI and the WPI-RES complex. The potential application of the complex in sarcopenia-related nutritional strategies was preliminarily considered, and the comparison might help guide the design of functional food ingredients.

## 2. Materials and Methods

### 2.1. Materials

Whey protein isolate (WPI, purity 90%) was purchased from Shanghai Yuanye Bio-Technology Co., Ltd. (Shanghai, China). Resveratrol (RES, purity 98%) was obtained from Zhejiang Xinhe Biotechnology Co., Ltd. (Ningbo, China). Anhydrous ethanol, anhydrous disodium hydrogen phosphate, anhydrous sodium dihydrogen phosphate, sodium bicarbonate, ethylenediaminetetraacetic acid (EDTA), 1,1-diphenyl-2-picrylhydrazyl (DPPH), sodium hydroxide, hydrochloric acid and citric acid monohydrate were of analytical grade and purchased from Sinopharm Chemical Reagent Co., Ltd. (Shanghai, China). The Bradford protein assay kit (BL524A) was acquired via Biosharp Co., Ltd. (Hefei, China). 8-Anilino-1-naphthalenesulfonic acid (ANS) of analytical grade was obtained from Shanghai Aladdin Biochemical Technology Co., Ltd. (Shanghai, China). Simulated gastric fluid and simulated intestinal fluid were purchased from Beijing Leigen Biotechnology Co., Ltd. (Beijing, China). A 3500 Da cutoff dialysis bag and ultrapure water were used throughout the experiments.

### 2.2. Preparation of WPI-RES Non-Covalent Complex

In this work, molar ratio calculations were performed using β-lactoglobulin (molecular weight: 18.3 kDa), the most abundant protein component in WPI. According to the ADPI Beta-lactoglobulin Standard v 2.0 (2023), β-Lg accounts for at least 70% of the total protein in WPI. This treatment served as a simplified approximation for complex preparation [10]. Following the method of Carson [11], WPI isolate was dissolved evenly in 0.1 M PBS (pH 7.0), after being agitated at 400 rpm and 25 °C for 2 h, the WPI solution was kept at 4 °C for 12 h to give a 500 μM protein solution. Resveratrol was first dissolved in absolute ethanol to prepare a 50 mM stock solution with the aid of brief sonication. An aliquot of this stock solution was then slowly added dropwise into 0.1 M PBS (pH 7.0) under constant stirring to obtain a final concentration of 500 μM. The working solution was freshly prepared and protected from light prior to use. The two were mixed at given molar ratios and stirred at 400 rpm for the reaction time in darkness at 25 °C. The mixture was dialyzed in a pre-treated bag against 0.1 M PBS (pH 7.0) at 4 °C for 48 h under dark conditions. During dialysis, 1 M NaOH or HCl solution was added to maintain the pH of the system. The dialysate was renewed every 24 h. Dialysis was terminated when the absorbance of the external dialysate at 300 nm approached the baseline value to ensure complete removal of unbound RES monomers. Samples were frozen at −80 °C for 24 h prior to 72 h lyophilization, producing powdered WPI-RES non-covalent complexes. The powder was stored in a desiccator protected from light until further use. Blank WPI controls were subjected to the same incubation, dialysis, and freeze-drying procedures without the addition of RES.

### 2.3. Optimization and Validation of Preparation Conditions for WPI-RES Complex

Single-factor experiments were conducted to evaluate the effects of pH (5.0, 6.0, 7.0, 8.0 and 9.0), reactant molar ratio (WPI:RES = 1:0.2, 1:0.6, 1:1, 1:1.4 and 1:1.8) and reaction time (1.0, 1.5, 2.0, 2.5 and 3.0 h) on the WPI-RES complex’s DPPH radical scavenging rate and protein digestibility. According to the single-factor results in Table S1, an L_9_(3^4^) orthogonal array design (with a blank column) was used with three factors: pH (A: 6.0, 7.0 and 8.0), reaction time (C: 1.5, 2.0 and 2.5 h) and molar ratio (D: 1:1.0, 1:1.4 and 1:1.8). The factor levels were shown in Table S2. Using the membership degree (Equation (1)) and weighted sum (Equation (2)) to calculate the comprehensive score, the optimal preparation conditions (pH 7.0, 1:1 molar ratio, and 1.5 h) were determined by range and variance analyses. A validation experiment was then conducted under these optimal conditions, where $\mu_{i}$ was the membership degree of the ith indicator, obtained by min–max normalization of the measured value $X_{i}$ ($X_{min}$ and $X_{max}$ were the minimum and maximum values across all samples); S was the composite score, defined as the weighted sum of $\mu_{DPPH}$ (membership degree of DPPH radical scavenging rate, weight 0.6) and $\mu_{PD}$ (membership degree of protein digestibility, weight 0.4).

(1) $$\mu_{i}=\frac{X_{i}-X_{min}}{X_{max}-X_{min}}$$

(2) $$S=\mu_{\mathrm{DPPH}}\times 0.6+\mu_{\mathrm{PD}}\times 0.4$$

### 2.4. Determination of DPPH Radical Scavenging Rate

Following the method of Han [12] with slight modifications, anhydrous ethanol was used to dissolve DPPH. at 1 mM and stored at 4 °C protected from light. Prior to use, it was diluted 10-fold to 0.1 mM. Sample solutions were prepared at 1 mg/mL. Absorbance readings of the listed mixtures were taken at 517 nm: 3.0 mL of 0.1 mM DPPH solution + 3.0 mL of distilled water (*A*_0_); 3.0 mL of 0.1 mM DPPH solution + 3.0 mL of sample (*A_i_*); and 3.0 mL of anhydrous ethanol + 3.0 mL of sample (*A_j_*). The DPPH radical scavenging rate (Equation (3)) was calculated as follows:

(3) $$\mathrm{DPPH}\;\mathrm{radical}\;\mathrm{scavenging}\;\mathrm{rate}\;(\%)=\left[1-\frac{A_{i}-A_{j}}{A_{0}}\right]\times 100$$

### 2.5. In Vitro Protein Digestibility

Based on the protocol of Xing [13] with adjustments, the complex powder was dissolved in ultrapure water to prepare a 5 mg/mL complex solution. An equal volume of artificial gastric fluid was added. The mixture was then incubated at 37 °C for 2 h with shaking, then adjusted to pH 6.8–7.0, mixed with an equal volume of simulated intestinal fluid, and shaken for another 2 h at 37 °C. Enzymes were inactivated by boiling for 10 min. Protein contents in pre-digestion and post-digestion samples were determined by Bradford assay, with ultrapure water as blank. Protein digestibility was calculated according to (Equation (4)), where *A_s_*, *A_b_* and *A*_0_ represented the supernatant protein content, blank digest protein content and initial protein content, respectively.

(4) $$\mathrm{Digestibility}\;(\%)=\frac{A_{s}-A_{b}}{A_{0}}\times 100$$

### 2.6. Amino Acid Content Determination

The WPI-RES complex underwent in vitro digestion following the procedure described above. Dialysis of the digest was conducted against distilled water at 25 °C for 2 h with stirring at 400 rpm using a 500 Da MWCO membrane, then concentrated by rotary evaporation and lyophilized. Amino acid analysis of the lyophilized powder followed GB 5009.124-2016. Briefly, 0.3500 g of sample was freeze-thawed in liquid nitrogen, the sample was then subjected to acid hydrolysis with a 10 mL volume of 6 M HCl supplemented with phenol. The hydrolysis proceeded at 110 ± 1 °C for 22 h under a nitrogen atmosphere. The hydrolysate was filtered, diluted to 50 mL, and filtered again through a 0.22 μm membrane for analysis using an L-8900 automatic amino acid analyzer (Hitachi, Tokyo, Japan).

### 2.7. UV-Vis Absorption Spectroscopy

Following the method of Ma [14] with modifications, WPI-RES non-covalent complex and WPI solutions were prepared at concentrations of 0.1, 0.5 and 1.0 mg/mL. UV-Vis spectra of the solutions were recorded over a wavelength range of 190 to 750 nm with a resolution of 0.1 nm using a Thermo Fisher Scientific spectrophotometer (Waltham, MA, USA). Each scan was performed in triplicate. For structural characterization, 300 and 700 μM in addition to 500 μM were selected to form a concentration gradient around the optimum. This design allowed us to examine how RES concentration affects WPI conformation and to gain a more comprehensive understanding of the interaction mechanism.

### 2.8. Intrinsic Fluorescence Spectroscopy

Following the fluorescence analysis system established by Michael [15], WPI and WPI-RES complex solutions at 0.05, 0.1, and 0.2 mg/mL were prepared. One milliliter of each solution was placed in a quartz cuvette with four optical windows. Emission spectra in the range of 300–450 nm were collected using an F-4500 fluorescence spectrophotometer (Hitachi, Tokyo, Japan) with the following instrument parameters: excitation wavelength 280 nm, excitation and emission slit widths 5.0 nm, scanning speed 1200 nm/min, response time 8.0 s and photomultiplier tube voltage 750 V. All samples were measured in triplicate and the average values were used for data analysis.

### 2.9. Synchronous Fluorescence Spectroscopy

Following the method of Yu [16], 1 mL of 0.05, 0.1, and 0.2 mg/mL WPI-RES non-covalent complex or WPI solutions were placed in quartz cuvettes. Tyrosine and tryptophan residues were analyzed by synchronous fluorescence at Δλ of 15 nm and 60 nm, respectively. The scan range was set from 200 to 350 nm with a rate of 1200 nm/min. Measurements were made at 5.0 nm slit width, 700 V, and 0.50 s response time. The average of three repeated scans was recorded.

### 2.10. Three-Dimensional (3D) Fluorescence Spectroscopy

Following the method of Allahdad [17] with preliminary experiments to adjust parameters, ultrapure water was used as a blank control. One milliliter of WPI-RES non-covalent complex or WPI solutions at concentrations of 0.05, 0.1 and 0.2 mg/mL was placed in quartz cuvettes. 3D fluorescence spectra were collected via the 3D-scan mode. Fluorescence spectra were recorded with excitation/emission wavelengths of 200–350 nm and 200–500 nm, with a scanning speed of 1200 nm/min and a sampling interval of 2 nm. The slit width was set to 5.0 nm and the voltage was 700 V.

### 2.11. Fourier Transform Infrared (FTIR) Spectroscopy

Following the method of Jiang [18] with slight adjustments, the secondary structures of the WPI-RES non-covalent complex and WPI alone were analyzed by an IRPrestige-21 Fourier transform infrared spectrometer (Shimadzu Corporation, Kyoto, Japan). Accurately weighed lyophilized sample powder (2 mg) and KBr (200 mg) were ground together and pressed into a pellet. Spectra (400–4000 cm^−1^, 32 scans, 4 cm^−1^ resolution) were recorded and secondary structure changes were calculated by Peakfit 4.12 deconvolution of the amide I band (1600–1700 cm^−1^).

### 2.12. Surface Hydrophobicity Determination

Following the method of Alizadeh-Pasdar [19], the fluorescent probe ANS was employed to determine surface hydrophobicity. Treating the solutions of WPI or WPI-RES non-covalent complex (4 mL, 0.0125–0.1 mg/mL) with 8 mM ANS (20 µL) was followed by dark incubation for 15 min, after which fluorescence was measured using an F-4500 fluorometer (HITACHI, Tokyo, Japan) set to 390 nm (excitation) and 470 nm (emission), with a voltage of 500 V and slit widths of 5.0 nm.

### 2.13. Microstructure Observation of Complexes

Following the method of Wang [20] with slight modifications, approximately 1 mg each of the WPI-RES complex and WPI powder was mounted onto stubs with conductive adhesive using a JMS-6700F SEM (JEOL, Tokyo, Japan). The samples were observed at an accelerating voltage of 15 kV under different magnifications. Images were captured at 1500× and 500× magnification.

### 2.14. Molecular Docking Analysis

RES (PubChem CID: 445154) was prepared using Schrödinger Ligprep (OPLS_4), with stereoisomers and protonation states assigned via Epik. Protein preparation and energy minimization were performed with the Protein Preparation Wizard. Docking (Glide SP) used SiteMap-predicted pockets, with a 10 Å inner box centered on the site centroid. Up to 10 conformations per ligand were generated with post-docking minimization, and the lowest-score pose was selected for subsequent analysis. The system was considered converged when the protein backbone RMSD reached a plateau with stable fluctuations around the average and the total energy remained stable over time [21].

### 2.15. Molecular Dynamics (MD) Simulation

Protonation states of protein residues were assigned with PROPKA 3.0, and crystallographic waters were retained. The complex was built in Schrödinger System Builder (OPLS_4, TIP3P solvation) and placed in a cubic water box with a 10.0 Å buffer, then neutralized. After energy minimization (50,000 steps), NVT and NPT equilibrations (50,000 steps each) were performed with heavy-atom restraints at 300 K and 1 atm. Post-simulation analyses (Schrödinger 2023) included RMSD, RMSF, ROG, secondary structure, interactions, and MM-GBSA binding energies. Molecular dynamics simulations were performed in triplicate using independently generated initial velocities to ensure reproducibility.

### 2.16. Statistical Analysis

All experiments were performed in triplicate (three parallel replicates). The experimental data were organized and graphically processed using Origin 2025 software. Statistical analysis was performed with SPSS 27.0, and Duncan’s multiple range test was applied for mean comparisons at a significance level of *p* < 0.05.

## 3. Results and Analysis

### 3.1. Preparation of the Non-Covalent Complex

**Figure 1:**
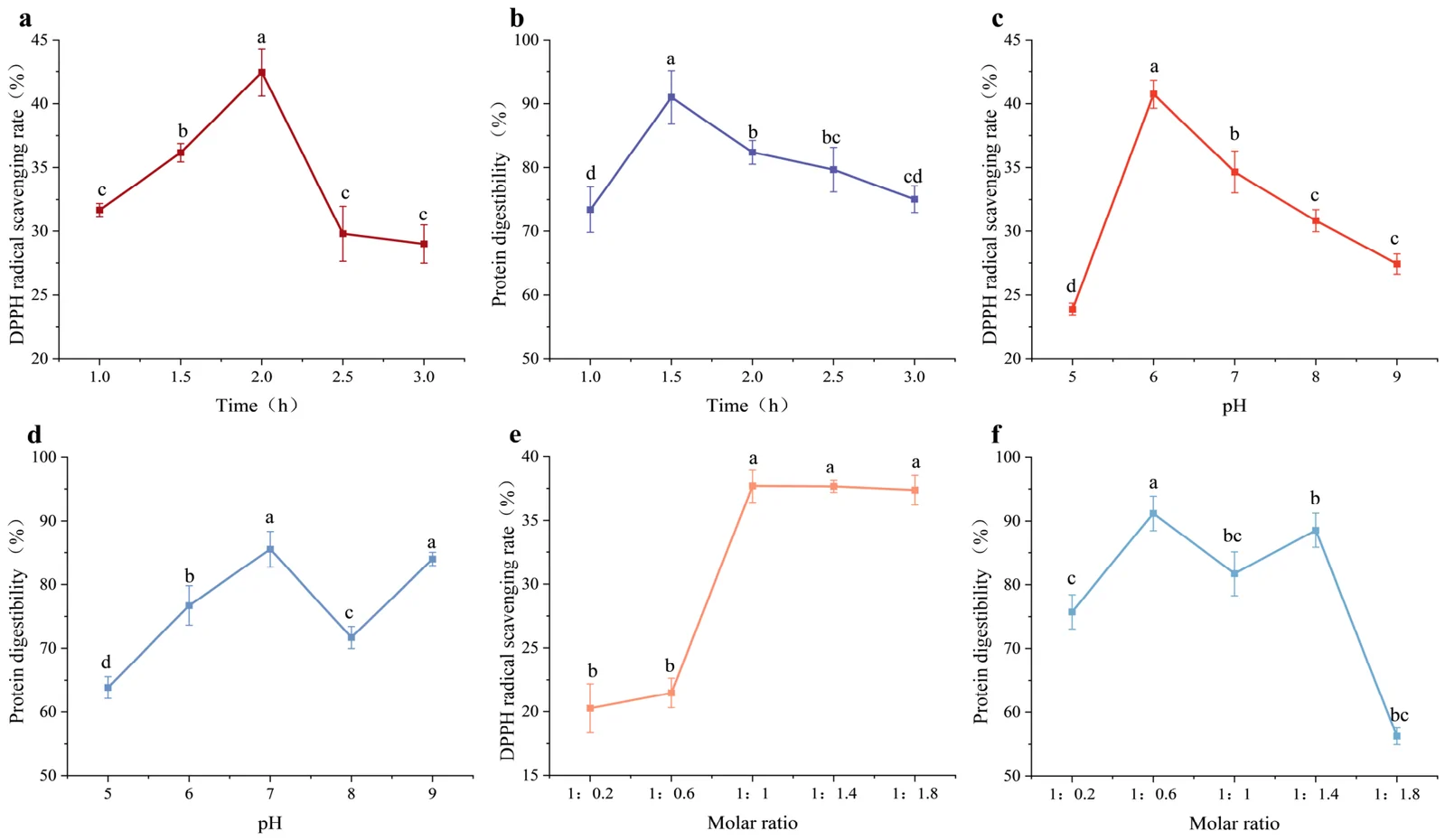
Effect of time on inhibition of DPPH (**a**) and in vitro protein digestibility (**b**); effect of pH on inhibition of DPPH (**c**) and in vitro protein digestibility (**d**); effect of molar ratio on inhibition of DPPH (**e**) and in vitro protein digestibility (**f**) of WPI-RES. Note: different lowercase letters in the figure indicate significant differences (*p* < 0.05).

Protein digestibility and DPPH radical scavenging rate first increased and then decreased with reaction time. As shown in Figure 1a,b, with prolonged reaction time, protein digestibility reached its maximum of 90.99 ± 4.15% at 1.5 h, while the DPPH radical scavenging rate peaked at 42.45 ± 1.86% at 2 h. As shown in Figure 1c,d, with increasing pH, the DPPH radical scavenging rate attained its highest value of 40.73 ± 1.09% at pH 6.0, whereas protein digestibility rose again to 83.98 ± 1.06% at pH 9.0. As shown in Figure 1e,f, protein digestibility with increasing RES proportion reached 91.20 ± 2.72% and 88.55 ± 2.70% with molar ratios respectively at 1:0.6 and 1:1.4, whereas DPPH scavenging continuously climbed to the 1:1 ratio before leveling off. Such observations imply that partial protein unfolding under moderate reaction conditions enhances accessibility of cleavage sites [22]. Excessive reaction conditions might induce protein cross-linking and aggregation, which impair radical-scavenging activity [23]. Optimization of the WPI-RES complex preparation was performed via range and variance analyses, with the comprehensive score as the criterion. As shown in Table S3, pH exhibited the most significant influence on the overall performance of the complex, whereas the effects of reaction time and molar ratio were relatively limited under the tested conditions. In summary, the optimal preparation parameters for the WPI-RES complex were determined as pH 7.0, a WPI:RES molar ratio of 1:1.0, and a reaction time of 1.5 h.

When prepared under these optimal conditions, the complex exhibited a DPPH radical scavenging rate of 52.41 ± 0.58% and a protein digestibility of 83.60 ± 0.50%. Compared with 48.67 ± 0.65% and 82.21 ± 0.88% for WPI alone, WPI-RES non-covalent complex showed significantly greater DPPH scavenging than WPI alone, with a relative increase of 21.66% (*p* < 0.05), whereas protein digestibility did not differ significantly from that of WPI (*p* > 0.05). Taken together, the binding of RES to WPI enhanced the DPPH radical scavenging activity of the complex system without significantly compromising the digestibility of the protein, which was favorable for the anti-sarcopenic effect of the complex.

### 3.2. Amino Acid Content

Following acid hydrolysis, the amino acid compositions of WPI and WPI-RES complex are given in Table 1. According to the FAO/WHO (2007) ideal protein standard, the essential amino acids of WPI and WPI-RES complex accounted for 41.5% and 38.9%, respectively. The values were consistent with the FAO/WHO reference pattern, indicating that the amino acid profile of WPI remained nutritionally balanced after complexation with RES [24]. Branched-chain amino acids accounted for 21.4% and 18.6% of the amino acids in WPI and the complex, respectively, and hydrophobic amino acids for 37.6% and 35.4%, and glycine for 1.8% and 1.7%, respectively. Branched-chain amino acids served as key substrates for muscle protein synthesis [25]; hydrophobic amino acids could persistently exert antioxidant activities such as scavenging free radicals and inhibiting lipid peroxidation, which might effectively alleviate oxidative stress damage during sarcopenia [26], and glycine’s anti-inflammatory and antioxidant activities could still be realized, thereby preserving muscle mass and reducing atrophy [27].

The reduced amino acid release likely reflected non-covalent WPI-RES binding, which shielded protease active sites and suppressed free amino acid liberation [28]. This was consistent with the literature reporting that protein–phenolic interactions could lead to reduced amino acid content [29]. Also, relevant experiments demonstrated that complexation of WPI with chlorogenic acid (CQA) significantly decreased free amino groups and several individual amino acids, which aligned with the observations in the present study [30].

In conclusion, although the absolute amino acid contents in the WPI-RES complex were lower, the nutritionally balanced profile was largely preserved. Meanwhile, the complex exhibited significantly improved DPPH radical scavenging capacity compared with native WPI. However, given the heterogeneous nature of sarcopenia and the absence of in vivo validation in this study, the actual efficacy of the complex in sarcopenia management warrants further investigation [31].

### 3.3. UV-Vis Absorption Spectroscopy

**Figure 2:**
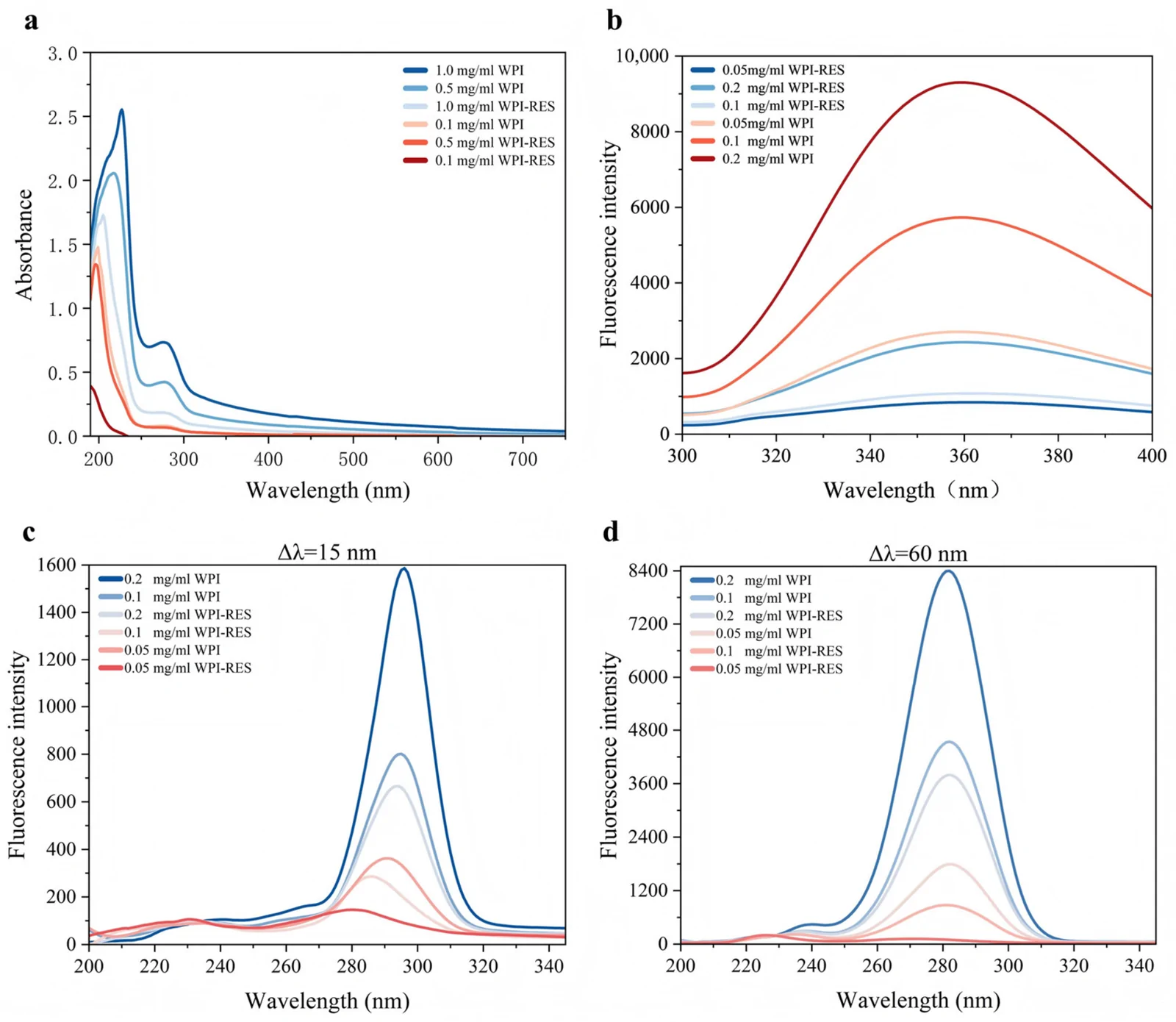
Ultraviolet–visible spectra of WPI and WPI-RES non-covalent complex (**a**); intrinsic fluorescence spectra of WPI and WPI-RES non-covalent complex (**b**); synchronous fluorescence spectra of WPI and WPI-RES non-covalent complex at Δλ = 15 nm (**c**) and Δλ = 60 nm (**d**).

As shown in Figure 2a, the absorption peak of WPI underwent a red shift with increasing concentration; WPI at 1.0, 0.5, and 0.1 mg/mL displayed distinct absorption peaks at 227, 218, and 199 nm, respectively. At the same concentrations, the WPI-RES complex showed a blue shift and a hypochromic effect relative to WPI alone. The strong absorption peaks of WPI and its complex originated mainly from the π → π* electronic transition of the peptide bond C=O, with additional contributions from aromatic amino acid side chains and protein secondary structures. Around 270 nm, a subtle absorption peak accompanied by a hypochromic effect was observed for all samples. Compared with WPI, the WPI-RES complex exhibited a hyperchromic effect and a blue shift in this region. Harimana et al. [32] reported that the binding of soybean protein with RES also induced a blue shift in both the 200–230 nm peptide bond region and the 270–280 nm aromatic region. RES was inserted into the hydrophobic cavity of WPI via hydrophobic interactions, wrapping the originally solvent-exposed aromatic amino acid residues into a hydrophobic environment, thereby reducing the polarity of the chromophore microenvironment and increasing the electronic transition energy. For the WPI–RES complex, insertion of RES into the hydrophobic cavity limited the electronic transitions of aromatic residues and produced a hypochromic effect. A similar phenomenon has been reported for the binding of the flavonoid quercetin to human serum albumin, where restricted electronic transitions within the binding pocket also gave rise to a hypochromic effect [33].

### 3.4. Intrinsic Fluorescence Spectroscopy

As shown in Figure 2b, all WPI samples displayed a strong characteristic emission peak at 359 nm, and the fluorescence intensity decreased progressively with decreasing protein concentration, indicating that the signal predominantly originated from the densely packed tryptophan residues within the protein [34]. Compared with WPI alone, the WPI-RES complex at the same concentrations exhibited fluorescence quenching. The WPI-RES complex showed concentration-dependent red shifts of 3 nm, 2 nm and 1 nm at 0.05, 0.1 and 0.2 mg/mL, respectively, relative to the corresponding WPI samples. Geng et al. [35] confirmed that the binding of flavonoids to β-lactoglobulin (β-Lg) induced a slight red shift of the characteristic emission peak of tryptophan residues, which was attributed to the specific binding of the polyphenol to Trp19, leading to increased polarity of the microenvironment surrounding the tryptophan residue. The fluorescence quenching and small red shift indicate that RES perturbed internal chromophores, reducing radiative efficiency, while the hydrophobic core remained intact and Trp residues stayed in the cavity. RES likely binds near Trp, causing slight local polarity increase without disrupting overall conformation [36].

### 3.5. Synchronous Fluorescence Spectroscopy

As shown in Figure 2c, at Δλ = 15 nm, the synchronous fluorescence intensity of the WPI-RES complex decreased markedly compared with that of WPI at the same concentration, and all maximum emission wavelengths exhibited a blue shift. The blue shifts for the 0.2, 0.1, and 0.05 mg/mL groups were 1, 9, and 11 nm, respectively. These results indicated that RES bound to tyrosine residues of WPI through hydrophobic interactions, further reducing the polarity of the microenvironment around these residues and embedding them into a more hydrophobic environment, which accounted for the observed blue shift [37]. As shown in Figure 2d, at Δλ = 60 nm, only the 0.05 mg/mL WPI-RES non-covalent complex group displayed a significant blue shift; the maximum emission wavelengths of the other groups remained stable at approximately 283 nm. This observation was consistent with the findings of Mohammadi [38]. RES binding slightly changed the WPI tertiary structure, perturbing the hydrophobic network around tryptophan and lowering quantum yield, thus enhancing quenching. This directly reflected the high sensitivity of the tryptophan region to RES binding. The above results collectively suggested that the interaction between RES and WPI did not act on a single type of aromatic amino acid residue; rather, it affected the microenvironments of both tyrosine and tryptophan residues through multi-site binding. The binding of RES to WPI disrupted the balance between intramolecular hydrophobic interactions and hydrogen bonds, leading to a transition of the protein conformation from a compact to a looser state [39].

### 3.6. Three-Dimensional Fluorescence Spectroscopy

**Figure 3:**
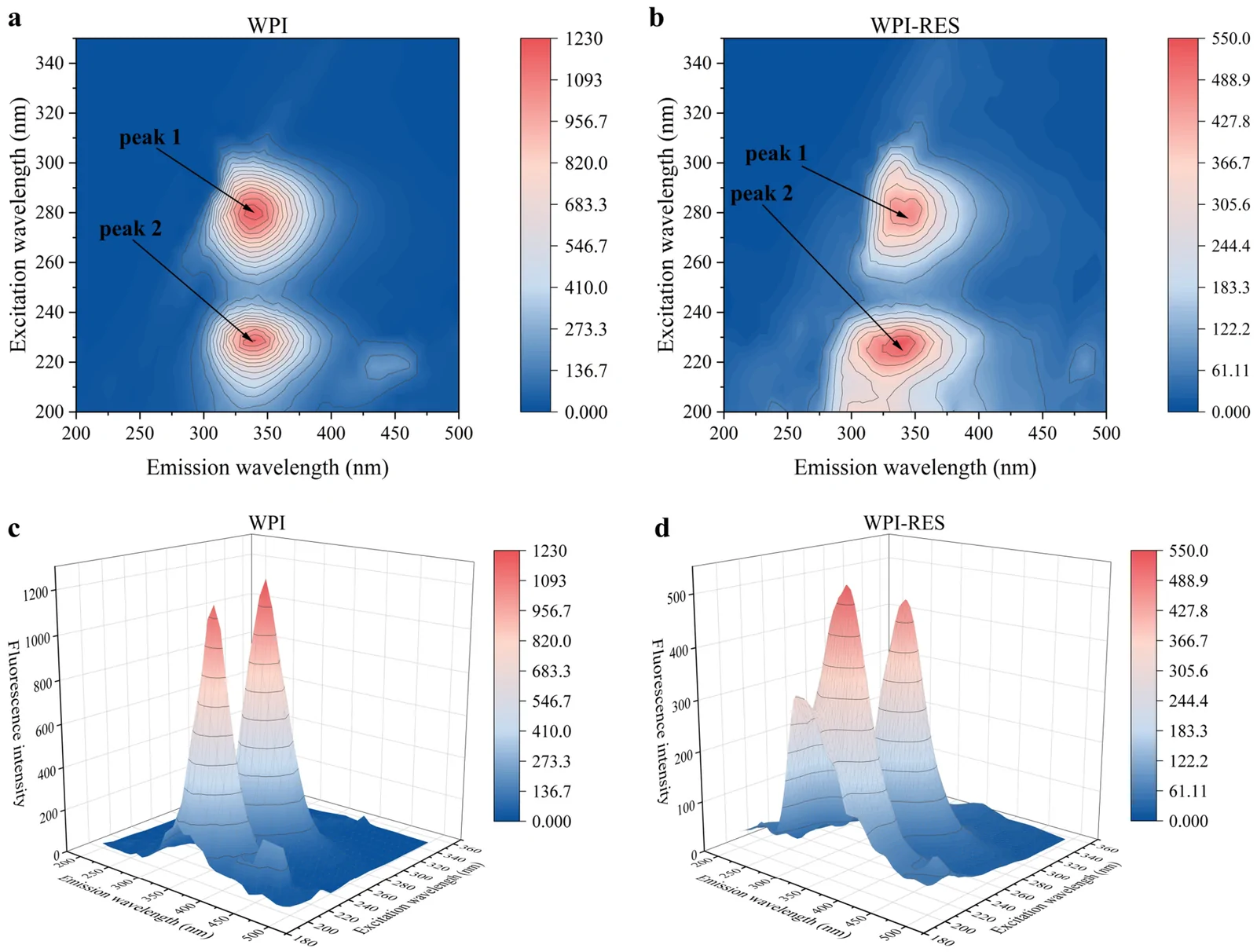
3D fluorescence spectra of WPI (**a**) and WPI-RES non-covalent complex (**b**); 3D fluorescence contour map of WPI (**c**) and WPI-RES non-covalent complex (**d**).

According to Figure 3, both WPI and WPI-RES non-covalent complex displayed two characteristic fluorescence peaks: Peak 1 primarily reflected the intrinsic fluorescence of tryptophan and tyrosine residues, while Peak 2 represented the fluorescence characteristic of the polypeptide backbone. As summarized in Table 2, compared with WPI, the fluorescence intensities of both peaks in the WPI-RES complex decreased significantly. The quenching rate of Peak 1 was 59.35%, accompanied by a 10 nm red shift. In conjunction with the synchronous fluorescence results, this quenching efficiency confirmed that tyrosine and tryptophan residues were the key binding sites for RES and participate in the hydrophobic interactions or hydrogen bonding within the complex, which was in agreement with the conclusions of Cheng et al. [40]. The quenching rate of Peak 2 was 52.72%, with no observable red or blue shift. This suggested that RES molecules were inserted between the WPI polypeptide chains via hydrophobic interactions or hydrogen bonding, disrupting the original hydrogen bond network maintaining the secondary structure and thereby altering the electronic transition environment of the peptide bonds, resulting in a marked decrease in fluorescence intensity. In a comparison of the quenching extents of the two peaks, the higher quenching rate of Peak 1 (59.35%) confirmed that aromatic residues were the core binding sites for RES, while the significant response of Peak 2 indicated that the binding interaction was not confined to surface residues; instead, it penetrated into the protein interior and induced global conformational changes. Similar changes in three-dimensional fluorescence spectra have been reported for other protein–polyphenol complexes, suggesting that alterations in the microenvironment of aromatic residues contributed to the conformational stability of the complex [41].

### 3.7. FTIR

**Figure 4:**
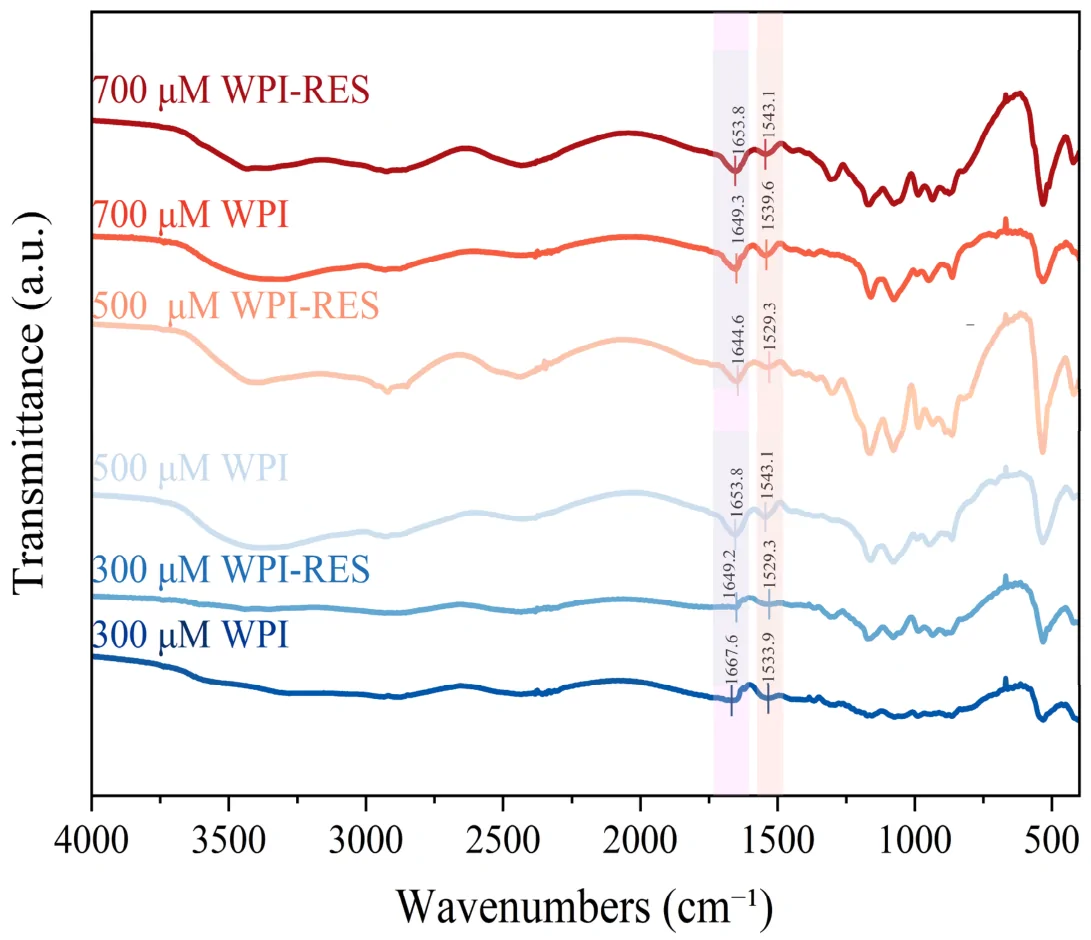
FTIR spectra of WPI and WPI-RES.

From Figure 4, WPI and the WPI-RES complex exhibited characteristic vibrational bands in the amide region. For WPI, the amide I band of 300, 500 and 700 μM appeared at 1667.6, 1653.8, and 1649.3 cm^−1^, respectively. After complexation with RES, the amide I band of the 300 and 500 μM groups red-shifted to 1649.2 and 1644.6 cm^−1^, respectively, whereas that of the 700 μM group blue-shifted to 1653.8 cm^−1^. This shift was attributed to changes in the microenvironment of the polypeptide C=O stretching vibrations, where alterations in the bond force constant and electron cloud density led to a systematic shift, similar FTIR spectral changes have been systematically reported for other non-covalent protein–polyphenol complexes [42]. The amide II band of WPI was observed at 1533.9, 1543.1, and 1539.6 cm^−1^. Upon complexation, both the 300 and 500 μM groups showed a red shift to 1529.3 cm^−1^, while the 700 μM group displayed a blue shift to 1543.1 cm^−1^. This could be attributed to intermolecular hydrogen bonding between protein amino groups and RES phenolic hydroxyls, which modified the coupling of N-H bending and C-N stretching vibrations [43]. A shift of the amide II band was also observed in a study of the whey protein isolate–resveratrol complex and was attributed to hydrogen bonding interactions [44]. Furthermore, in the saturated C-H stretching region (3000–2800 cm^−1^), the peak intensity of the WPI-RES complex was slightly enhanced and broadened compared with that of pure WPI, indicating hydrophobic interactions between the aromatic rings of RES and the hydrophobic side chains of WPI. Hydrophobic forces have been recognized as one of the major driving forces for resveratrol binding to whey protein [45], which further promoted the formation of the non-covalent complex. In the O-H/N-H stretching region (3200–4000 cm^−1^), the band of the complex was broader and more flattened than that of WPI, providing additional evidence for the formation and expansion of intermolecular hydrogen bond networks between RES and WPI [46], which strongly supported the stability of the non-covalent complex.

**Figure 5:**
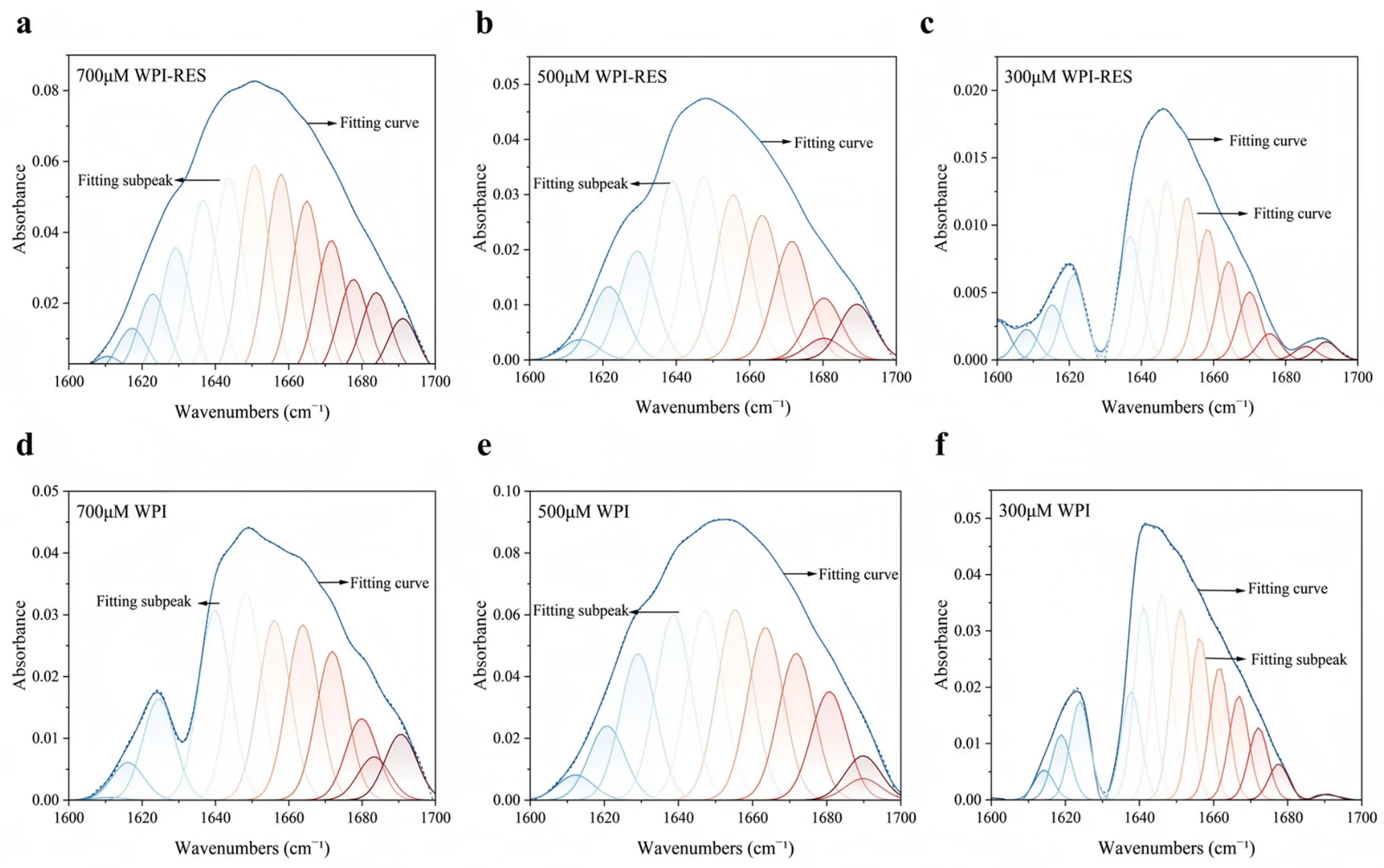
Curve-fitted amide I region of WPI and WPI-RES non-covalent complex. (**a**) 700 μM WPI-RES; (**b**) 500 μM WPI-RES; (**c**) 300 μM WPI-RES; (**d**) 700 μM WPI; (**e**) 500 μM WPI; (**f**) 300 μM WPI.

As shown in Table 3 and Figure 5, at 300 μM RES, non-covalent binding with RES led to a significant increase in β-sheet content, while the α-helix content slightly decreased and the β-turn content significant decreased, suggesting that RES induced these secondary structures into β-sheets, transforming the protein from a disordered state into a compact, ordered complex. At low concentrations, RES inserted into the flexible regions of the protein, accompanied by optimization of the intramolecular hydrogen bond network, which rendered the local structure more compact [47]. In the 500 and 700 μM systems, the α-helix content increased significantly, whereas random coil and β-turn contents decreased markedly. This was due to stronger intermolecular synergy at higher concentrations and cooperation among WPI components. As demonstrated by Yin et al. [48], the synergistic non-covalent interactions and disulfide bonds among whey protein constituents played a crucial role in stabilizing the protein–resveratrol complex, thereby leading to a more ordered and rigid secondary structure. This provided a structural basis for the overall stabilization of the secondary structure.

### 3.8. Surface Hydrophobicity

**Figure 6:**
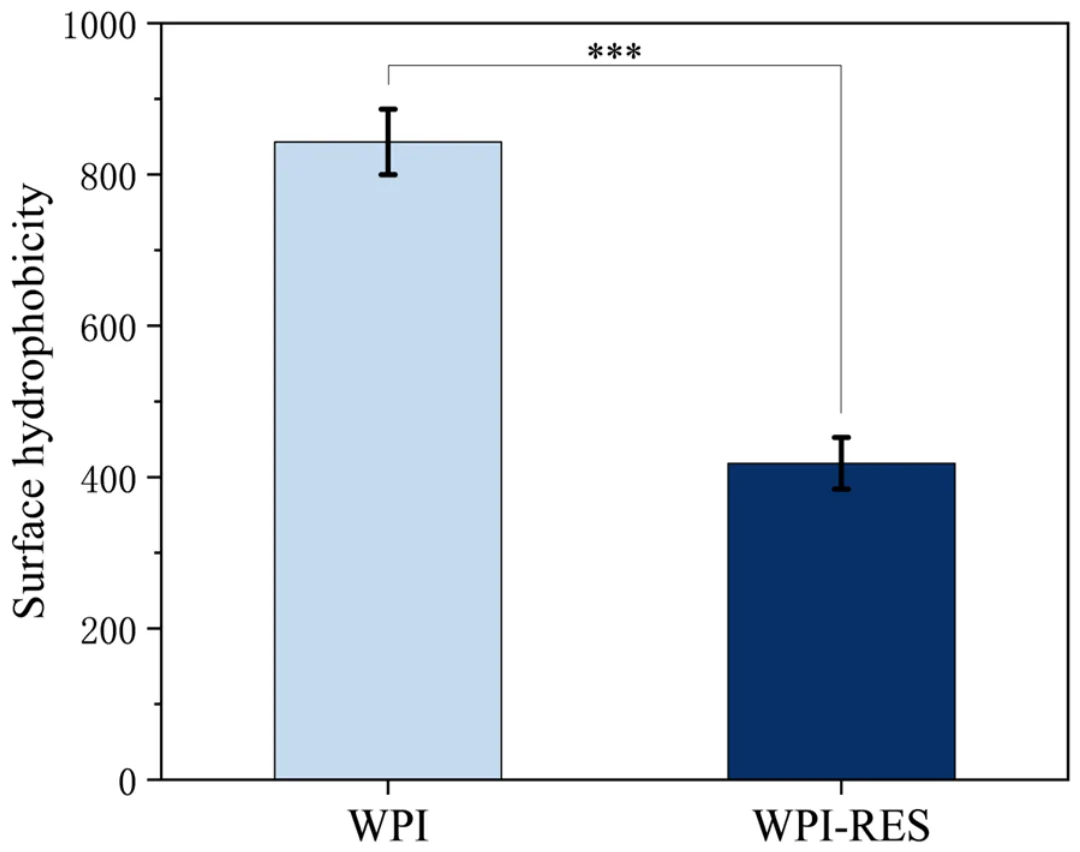
Surface hydrophobicity of WPI and WPI-RES non-covalent complex. Note: Asterisk (***) indicates statistically significant differences between the WPI and WPI-RES non-covalent complex (*p* < 0.05), n = 3.

The surface hydrophobicity directly reflected the regulation of protein conformation by the interaction between RES and WPI. According to Figure 6, the surface hydrophobicity of WPI-RES non-covalent complex exhibited a significantly lower value than WPI (*p* < 0.05). This change was highly consistent with the conformational rearrangement and altered hydrophobic residue microenvironment observed in the 3D fluorescence spectra. A plausible explanation was that RES, rich in hydrophobic aromatic rings, preferentially bound to the solvent-exposed hydrophobic residues on the WPI surface via hydrophobic interactions, thereby shielding these hydrophobic sites. This binding also rearranged WPI conformation globally, reburying exposed hydrophobic residues into the core. The combined effect of these two processes resulted in a significant decrease in surface hydrophobicity [49].

### 3.9. Microstructure of the Complex

**Figure 7:**
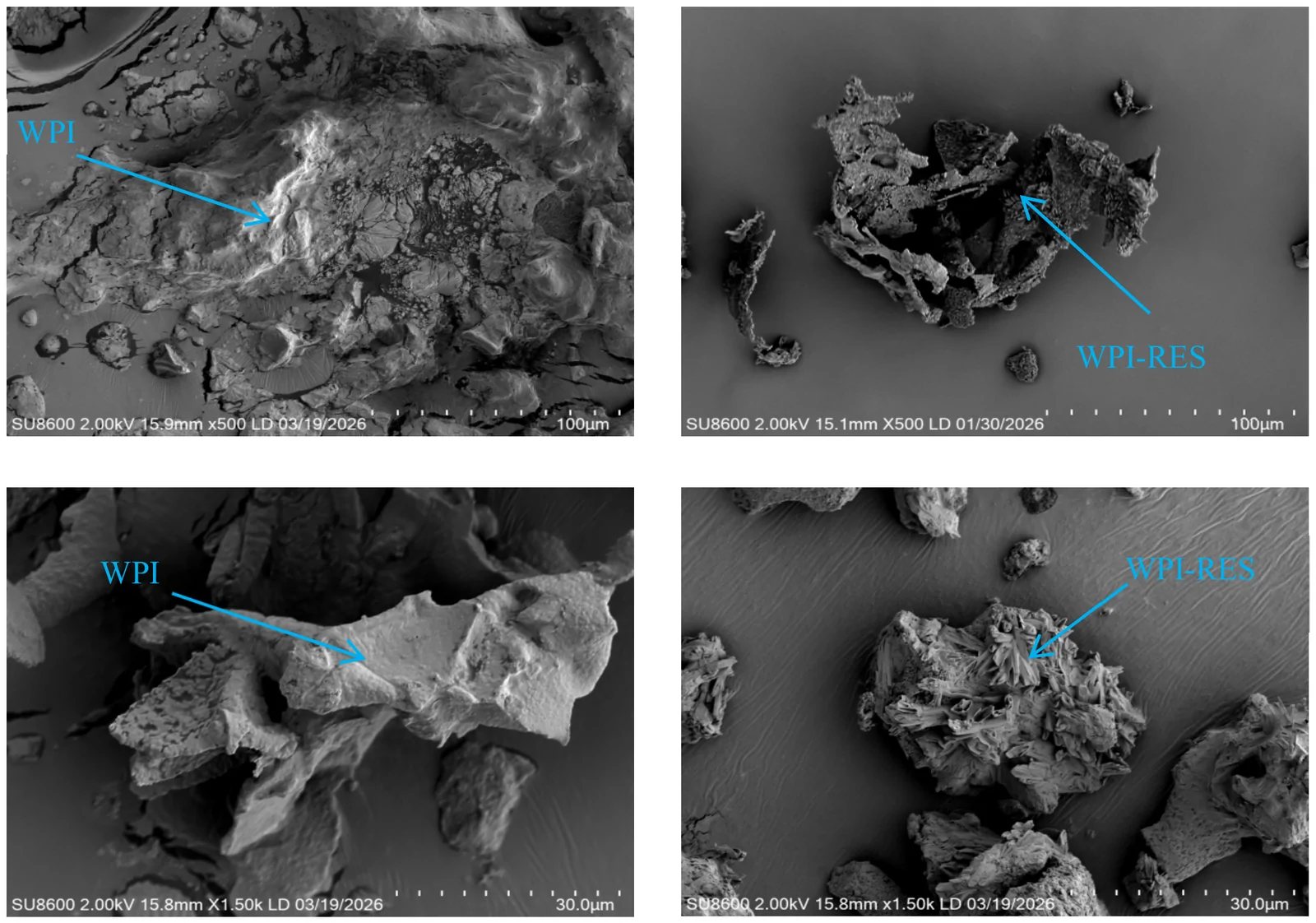
SEM images of WPI and WPI-RES non-covalent complex.

The SEM results were shown in Figure 7. Native WPI exhibited a continuous, dense, sheet-like aggregated structure with some surface wrinkles but an overall intact morphology. After complexation with RES, the microstructure of WPI-RES non-covalent complex changed remarkably: the original film-like structure was disrupted and transformed into a loose, porous, fragmented block-like morphology. At higher magnification, needle-like microcrystalline structures were observed adhering to the protein surface, and the inter-particle voids were markedly enlarged, this might be attributed to the intercalation of RES into the WPI intermolecular space through hydrophobic interactions, which disrupted hydrogen bonds and hydrophobic forces, thus creating a porous structure. Such a structure substantially increased the specific surface area of the complex, enhanced the encapsulation stability and antioxidant activity of RES, improved protein dispersibility, and strengthened the interaction between the complex and biological systems [50]. These morphological findings confirmed the spectroscopic conformational changes.

### 3.10. Molecular Docking

**Figure 8:**
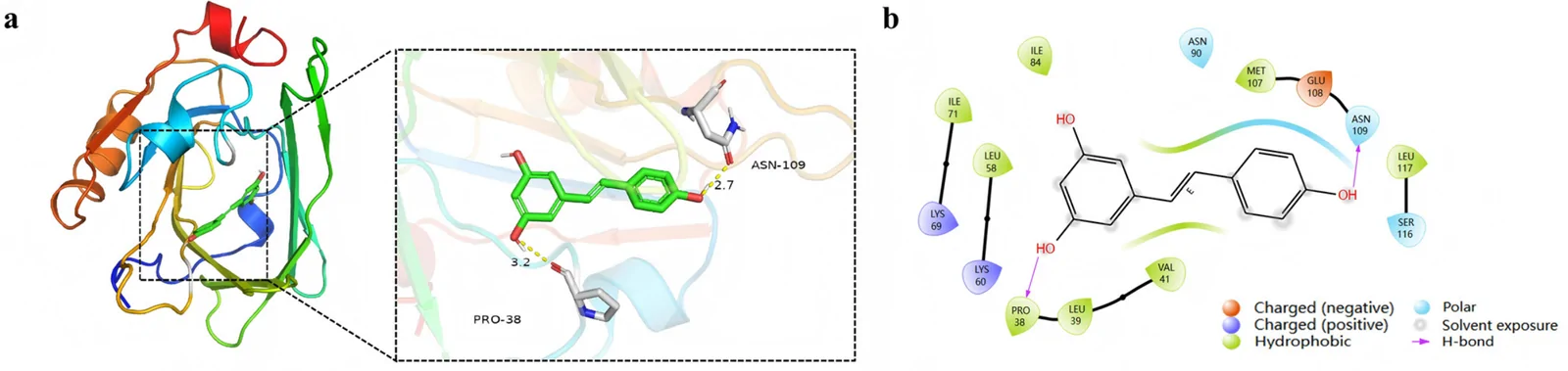
The best molecular docking analysis on interaction between f β-lactoglobulin and RES (**a**) and the 2D interaction diagram (**b**). The protein is shown as a cartoon, amino acid residues are represented as gray sticks, the small molecule (RES) is shown in green, and hydrogen bonds are illustrated by yellow dashed lines.

Since β-lactoglobulin (β-Lg) accounts for over 70% of WPI, which is the most abundant protein in whey protein isolate, it was selected as the representative model for molecular docking and dynamics simulations [51]. As shown in Figure 8, RES bound to the surface of β-Lg, with a docking score of −6.366 kcal/mol. This score was lower than the threshold for strong binding (−6.0 kcal/mol) [52], indicating a relatively strong binding stability between WPI and RES. In the complex, the RES molecule formed one hydrogen bond each with residues ASN109 and PRO38, and engaged in hydrophobic interactions with multiple surrounding amino acid residues. Hydrogen bonds and hydrophobic forces stabilized the β-Lg–RES complex, with hydrophobic interactions dominating RES binding to the protein surface. Meanwhile, docking analysis revealed that the β-lactoglobulin–resveratrol complex formed a stable non-covalent conformation, and the negative thermodynamic parameters (ΔG, ΔH, ΔS) indicated a spontaneous and exothermic binding process [53].

### 3.11. Molecular Dynamics Simulation

#### 3.11.1. Root Mean Square Deviation

**Figure 9:**
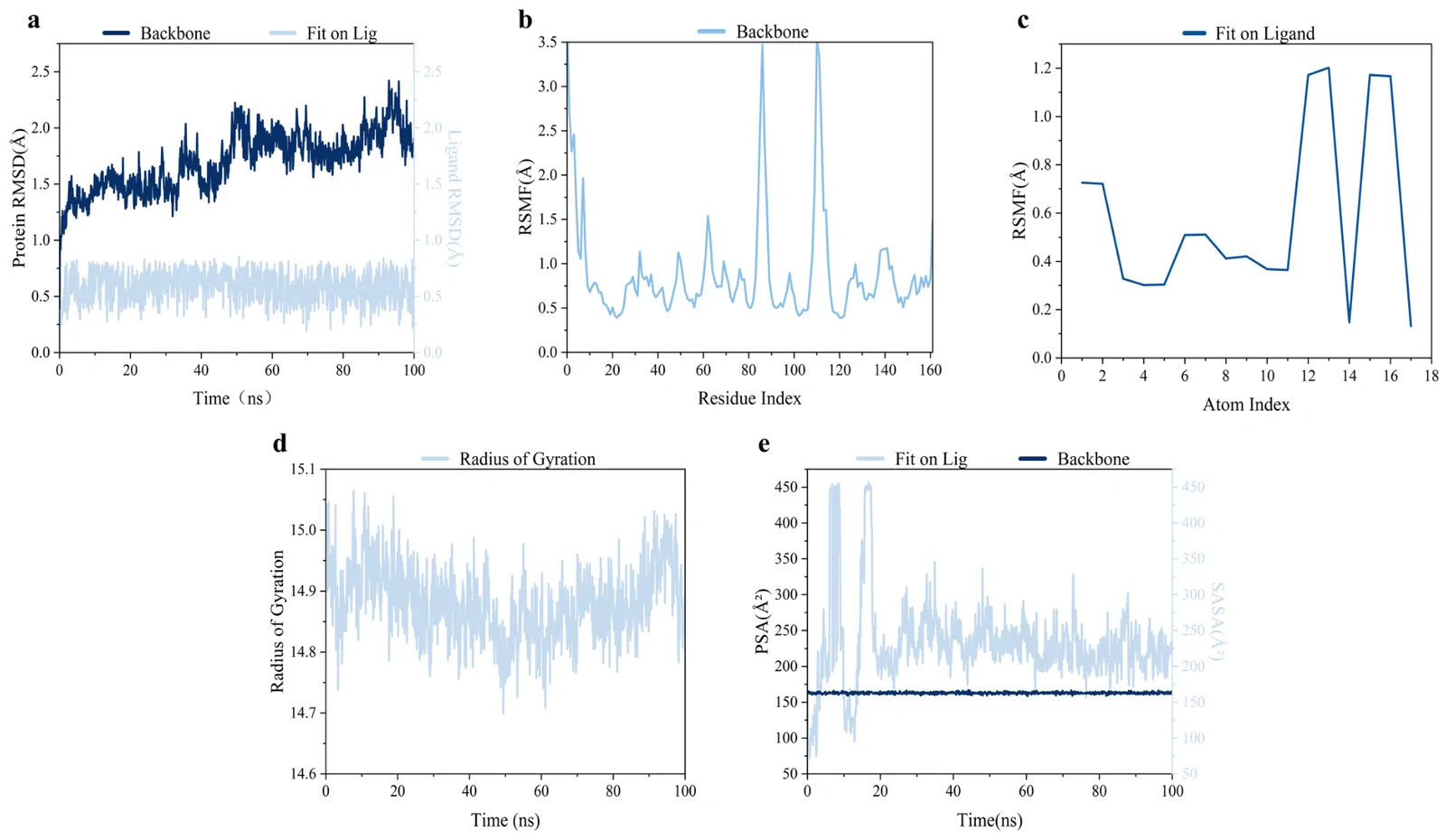
The RMSD of β-lactoglobulin and RES (**a**); RMSF of β-lactoglobulin (**b**) and RES (**c**) during 100 ns MD simulation; radius of gyration profile of the β-lactoglobulin backbone in the WPI-RES complex (**d**); time evolution of protein backbone solvent-accessible surface area (SASA) and ligand polar surface area (PSA) during 100 ns molecular dynamics simulation (**e**).

To gain deeper insight into the interaction mechanism, a 100 ns unrestrained molecular dynamics simulation was performed on the most stable binding conformation of RES with β-Lg. As shown in Figure 9a, the overall structure of the WPI-RES non-covalent complex remained stable without obvious dissociation or conformational rearrangement. During the initial phase of the simulation (0–20 ns), the RMSD of β-Lg increased; thereafter (20–100 ns), it stabilized and fluctuated within a narrow range of 1.5–2.4 Å, with no continuous upward trend, indicating that the protein backbone had reached dynamic equilibrium. The ligand RMSD remained consistently around 0.6 Å, demonstrating that its position in the binding pocket was highly stable. Hydrogen bonds formed likely restricted the conformational freedom of the protein and enhanced ligand stability. Throughout the simulation, both the RMSD and trajectory of RES confirmed the formation of a stable complex between RES and β-Lg [54].

#### 3.11.2. Root Mean Square Fluctuation

β-Lg and RES. According to Figure 9b, β-Lg RMSF values were relatively low, with increased fluctuations only observed in a few highly flexible loop regions. Notable conformational fluctuations occurred in the regions of residues 84–90 and 106–116, suggesting high flexibility during dynamics and a possible role as the ligand binding pocket. As shown in Figure 9c, the RMSF of the ligand RES remained consistently below 1.2 Å, confirming that the β-Lg-RES complex maintained a stable conformation and that the overall structural stability was satisfactory.

#### 3.11.3. Radius of Gyration

As shown in Figure 9d, the Rg value increased significantly during 0–20 ns and 50–100 ns. A larger Rg value indicated a more expanded system and a looser protein structure [55]. These increases were respectively attributed to thermal equilibration at the initial stage of the MD simulation [56] and to slight structural loosening caused by side-chain conformational rearrangements of residues near the binding site. During 20–50 ns, the Rg value gradually decreased, indicating that hydrogen bonds and hydrophobic interactions enabled β-Lg and RES to form a stable non-covalent complex, with the overall conformation becoming compact again. Throughout the entire simulation, the Rg value fluctuated within a small range of 14.7–15.1 Å, without any drastic rearrangement, indicating that the complex maintained good structural stability.

#### 3.11.4. SASA and PSA

As shown in Figure 9e, during the 100 ns MD simulation, the SASA value of RES showed sharp fluctuations in the 0–20 ns phase, indicating significant conformational rearrangement with a large initial exposure of hydrophobic regions, after which it rapidly stabilized. After 20 ns, the SASA value remained around 250 Å^2^, demonstrating that the ligand had attained a stable conformation with a well-buried hydrophobic core. This trend reflected a conformational transition of the ligand from an initial state to a stable state, where the hydrophobic core underwent dynamic adjustments and ultimately formed a more compact folded structure. A decrease in ligand SASA from an initially high value followed by stabilization is a common indicator of a stably bound conformation [57].

The polar surface area (PSA) reflected the interaction capacity of protein molecules with the solvent, as well as properties such as solubility and permeability. As shown in Figure 9e, throughout the 100 ns simulation, the PSA remained at approximately 160 Å^2^, indicating that the exposure of polar residues was highly consistent and the polar interface of the conformation was stable. This result demonstrated that after RES binding to β-Lg, the solvent-exposed area of the protein remained stable and no significant conformational rearrangement occurred, further confirming the structural stability of the complex. RES could perturb the microenvironment of β-Lg, enhance the solvation capacity of the protein, and reduce its hydrophobicity. Similarly, the stability of PSA in whey protein–EGCG complexes has been shown to be closely associated with their dispersibility and antioxidant activity in solution [58].

#### 3.11.5. Protein Secondary Structure Elements

**Figure 10:**
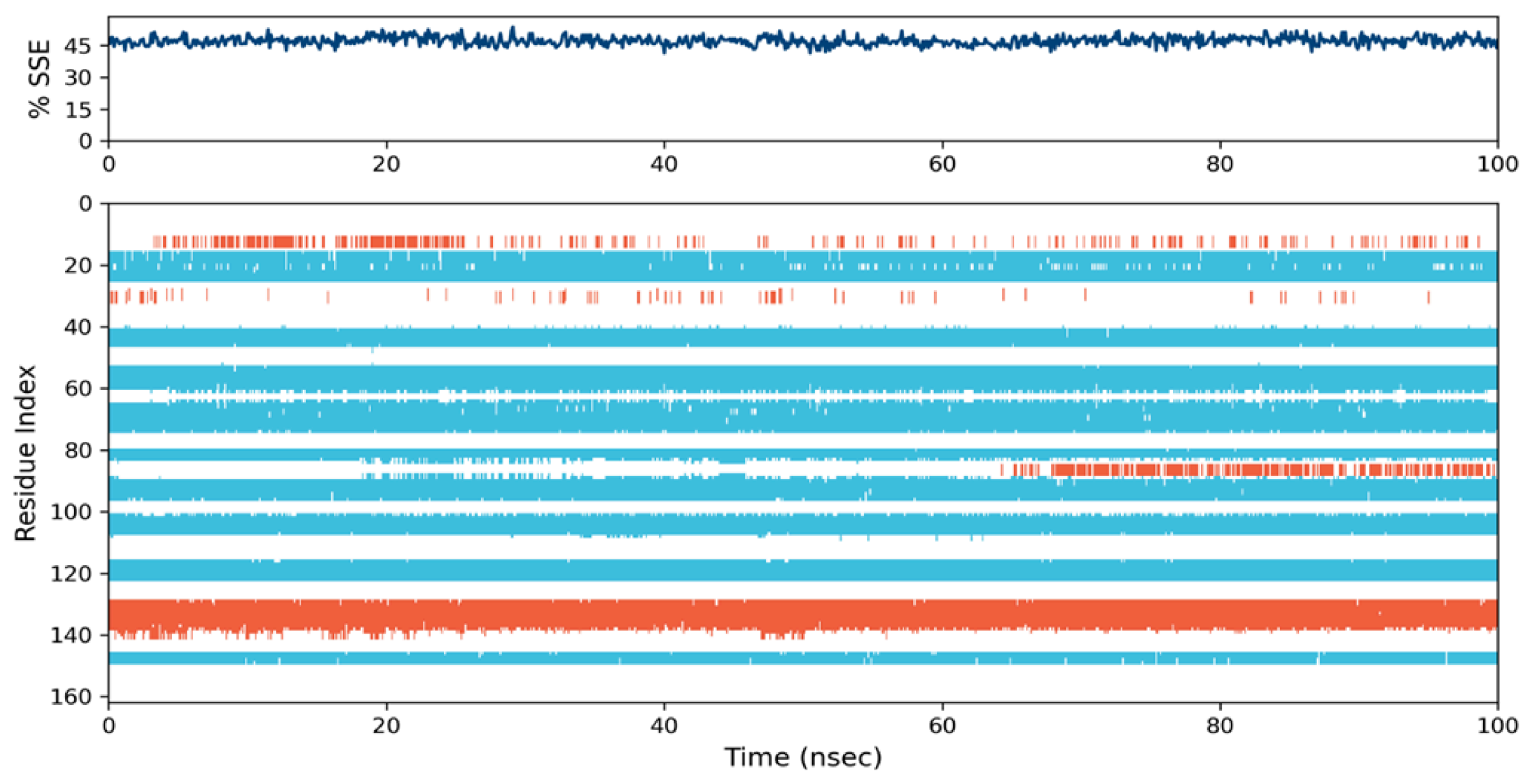
Secondary structure changes during 100 ns MD simulation.

After the 100 ns MD simulation, secondary structure analysis was performed. As shown in Figure 10, β-sheets (blue) and α-helices (orange) were the predominant secondary structure types. During the 60–100 ns period, a transition from β-sheet to α-helix occurred in the region around residues 80–90, which corresponded to a flexible loop region on the protein periphery. The protein’s secondary structure remained relatively stable throughout the rest of the simulation, with no significant structural changes. These results indicated that RES binding to β-Lg did not induce global unfolding or drastic rearrangement of the protein secondary structure; it only triggered a subtle conformational adjustment in a localized flexible region. This further confirmed the high conformational stability of the WPI-RES non-covalent complex [36].

#### 3.11.6. Molecular Dynamics Trajectory

**Figure 11:**
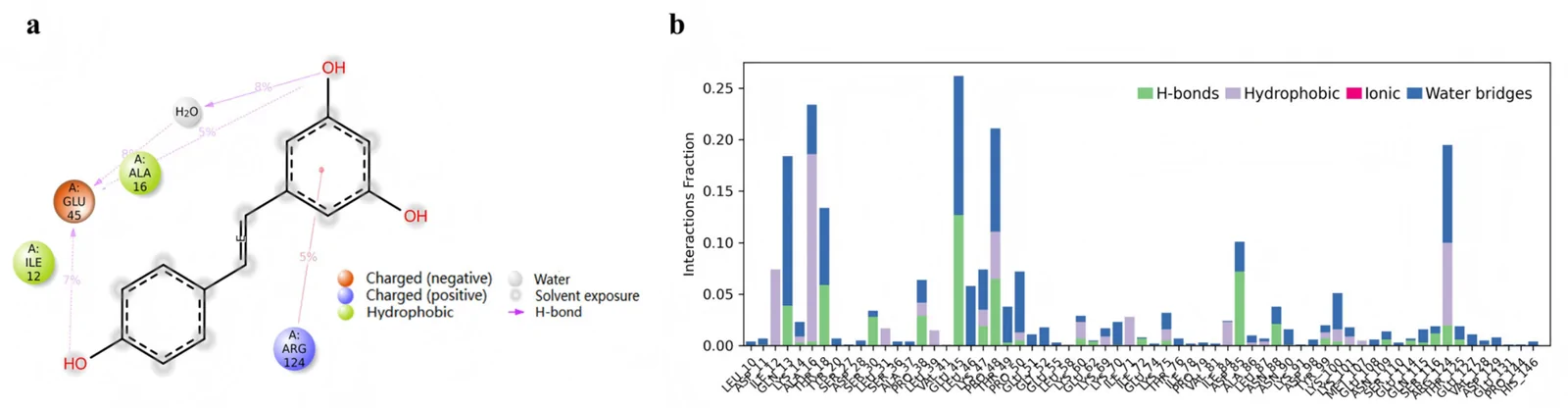
3D binding conformation and key interacting residues of ligand with protein (**a**); statistical fraction of different interaction types between each protein residue and ligand (**b**).

As shown in Figure 11, during the 100 ns MD simulation, hydrophobic interactions between the protein and the ligand played a dominant role throughout. Residues such as ILE12 and ALA16 engaged in hydrophobic contacts with the small molecule, serving as the core driving force for stabilizing the ligand within the binding pocket. Meanwhile, GLU45 formed a water-bridged hydrogen bond with the hydroxyl groups of RES. ARG124 formed a π-cation interaction with the small molecule. The binding mode of this complex represented a synergistic combination of hydrophobic and polar interactions, which jointly ensured the stable binding of the ligand to the protein. The key interacting residues predicted by molecular docking (ASN109, PRO38) differed from those obtained by MD simulation, since docking provided a static snapshot of ligand binding based on a rigid protein structure, whereas MD simulation allowed full conformational flexibility, enabling the ligand to relax and reposition toward a more thermodynamically favorable binding mode over time [59].

Analysis of the MD trajectory yielded the MM-GBSA binding free energy, calculated from the last frame of the equilibrated protein–ligand system. The binding free energy was −30.48 kcal/mol. This negative value confirms spontaneous binding and strong stability of the resulting complex. Consistent with previous reports, this value was interpreted as a qualitative indication of binding affinity rather than a precise thermodynamic quantity [60].

## 4. Conclusions

In this study, WPI and the WPI-RES complex were compared in terms of antioxidant activity and structure. Analysis combining spectroscopic data, docking, and MD simulations showed that RES bound primarily to the hydrophobic calyx of β-lactoglobulin in WPI. Hydrophobic interactions and hydrogen bonds drove this binding, with hydrophobic forces playing the dominant role. RES binding shifted the protein’s secondary structure toward a more compact, ordered conformation, reduced its surface hydrophobicity, and transformed its microstructure from a dense sheet-like form to a porous architecture. These structural changes enhanced the antioxidant capacity of the complex while preserving its protein digestibility. Long-term molecular dynamics simulations confirmed the high conformational stability of the complex. Overall, these findings highlighted the potential of WPI-RES complexes as functional ingredients for sarcopenia management. Further cellular and in vivo studies are still needed to translate these structural insights into practical nutritional interventions, which may support future research on sarcopenia management.

## Figures and Tables

**Table 1:** Amino acid content of WPI and WPI-RES non-covalent complex digested in vitro.

Name	WPI (g/100 g)	WPI-RES (g/100 g)
Asp	1.44	0.53
Thr	0.95	0.35
Ser	0.68	0.24
Glu	2.30	0.84
Gly	0.25	0.09
Ala	0.66	0.24
Cys	0.83	0.30
Val	0.81	0.21
Met	0.16	0.05
Ile	0.86	0.31
Leu	1.35	0.48
Tyr	0.76	0.53
Phe	0.52	0.25
Lys	0.98	0.36
His	0.23	0.08
Arg	0.39	0.14
Pro	0.95	0.36
Total	14.11	5.37

**Table 2:** 3D fluorescence spectrum results on the tertiary structures.

		Peak 1			Peak 2	
	Peak	Intensity	Change	Peak	Intensity	Change
	λ_ex_/λ_em_	F_0_	%	λ_ex_/λ_em_	F_0_	%
WPI	280/340	1227.45		230/340	1163.05	
WPI-RES	280/350	498.95	59.35	230/340	549.87	52.72

**Table 3:** The secondary structure contents of WPI and WPI-RES non-covalent complex.

Sample (μM)	Protein Secondary Structure Fractions (%)			
	β-sheets	Random coils	α-helices	β-turn
300 WPI	21.51 ± 0.18 ^e^	28.47 ± 0.89 ^a^	25.06 ± 0.12 ^ab^	24.96 ± 0.07 ^e^
300 WPI-RES	28.03 ± 0.36 ^c^	28.43 ± 0.47 ^a^	24.66 ± 0.55 ^b^	18.87 ± 0.62 ^f^
500 WPI	33.04 ± 0.11 ^a^	14.57 ± 0.42 ^c^	14.60 ± 0.80 ^d^	37.79 ± 0.15 ^b^
500 WPI-RES	31.15 ± 0.21 ^b^	10.81 ± 0.13 ^e^	22.57 ± 0.20 ^c^	35.47 ± 0.23 ^c^
700 WPI	26.90 ± 0.19 ^d^	16.80 ± 0.09 ^b^	14.58 ± 0.25 ^d^	41.72 ± 0.28 ^a^
700 WPI-RES	27.97 ± 0.24 ^c^	12.37 ± 0.24 ^d^	25.77 ± 0.02 ^a^	33.88 ± 1.04 ^d^

Note: Different lowercase letters in the figure indicate significant differences (*p* < 0.05).

## Data Availability

The original contributions presented in this study are included in the article and Supplementary Material. Further inquiries can be directed to the corresponding authors. The three-dimensional structure of bovine β-lactoglobulin (PDB ID: 3NPO) was obtained from the RCSB Protein Data Bank (RCSB PDB—3NPO: Bovine beta lactoglobulin unliganded form, accessed on 7 February 2026). The chemical structure of resveratrol (PubChem CID: 445154) was retrieved from the PubChem database (Resveratrol | C14H12O3 | CID 445154—PubChem, accessed on 7 February 2026).

## Supplementary Materials

The following supporting information can be downloaded at https://www.mdpi.com/article/10.3390/antiox15091096/s1; Table S1: Factors and levels used in single-factor experiments for WPI-RES complex. Table S2: The factors of orthogonal test on WPI-RES non-covalent complex. Table S3: Orthogonal test and range analysis of WPI-RES non-covalent complex.
