# Supplementary material for: Structural and Antioxidant Comparison Between Native WPI and WPI-Resveratrol Non-Covalent Complex
Source: Antioxidants (Basel). 2026 Aug 31;15(9):1096. doi: 10.3390/antiox15091096 (PMC13603859; doi:10.3390/antiox15091096)
Supplement: Supplementary file 1 [file antioxidants-15-01096-s001.zip › antioxidants-4496160-supplementary.pdf]

Table S1 Factors and levels used in single-factor experiments for WPI-RES complex.

No.	pH	Concentration Ratio（WPI:RES,molar ratio）	Time (h)
1	5.0	1∶0.2	1.0
2	6.0	1∶0.6	1.5
3	7.0	1∶1	2.0
4	8.0	1∶1.4	2.5
5	9.0	1∶1.8	3.0

Table S2 The factors of orthogonal test on WPI- RES non-covalent complex

No.	A	B	C	D
1	6.0	0	1.5	1:1.0
2	7.0	0	2.0	1:1.4
3	8.0	0	2.5	1:1.8

Note: Column B is a blank column used for error estimation.

Table S3 Orthogonal test and range analysis of WPI-RES non-covalent complex

NO.	A	B	C	D	DPPH Radical Scavenging Rate(%)	Protein Digestibility(%)	Comprehensive Score
1	1	1	1	1	52.590	81.520	0.781
2	1	2	2	2	49.570	79.490	0.620
3	1	3	3	3	45.980	76.210	0.406
4	2	1	2	3	46.470	85.620	0.653
5	2	2	3	1	49.010	82.690	0.676
6	2	3	1	2	45.660	90.530	0.742
7	3	1	3	2	38.890	77.540	0.174
8	3	2	1	3	39.950	74.040	0.129
9	3	3	2	1	36.490	80.260	0.151
K_1	1.807	1.609	1.652	1.609			
K_2	2.071	1.425	1.423	1.536			
K_3	0.454	1.299	1.257	1.188			
k_1	0.602	0.536	0.551	0.536			
k_2	0.690	0.475	0.474	0.512			
k_3	0.151	0.433	0.419	0.396			
R	0.539	0.103	0.132	0.140			
